# Regulation of candidalysin underlies *Candida albicans* persistence in intravascular catheters by modulating NETosis

**DOI:** 10.1371/journal.ppat.1012319

**Published:** 2024-06-17

**Authors:** Kuo-Yao Tseng, Yu-Tsung Huang, Yu-Ting Huang, Yu-Ting Su, An-Ni Wang, Wen-Yen Weng, Cai-Ling Ke, Yu-Chiao Yeh, Jhih-Jie Wang, Shin-Hei Du, Zi-Qi Gu, Wei-Lin Chen, Ching-Hsuan Lin, Yu-Huan Tsai

**Affiliations:** 1 Laboratory of Host–Microbe Interactions and Cell Dynamics, Institute of Microbiology and Immunology, National Yang Ming Chiao Tung University, Taipei, Taiwan; 2 Department of Laboratory Medicine, National Taiwan University Hospital, National Taiwan University College of Medicine, Taipei, Taiwan; 3 Department of Internal Medicine, National Taiwan University Hospital, National Taiwan University College of Medicine, Taipei, Taiwan; 4 Graduate Institute of Clinical Laboratory Sciences and Medical Biotechnology, National Taiwan University, Taipei, Taiwan; 5 Department of Biochemical Science and Technology, College of Life Science, National Taiwan University, Taipei, Taiwan; 6 Program in Molecular Medicine, National Yang Ming Chiao Tung University and Academia Sinica, Taipei, Taiwan; 7 Center for Molecular and Clinical Immunology, Chang Gung University, Taoyuan, Taiwan; University of California Los Angeles David Geffen School of Medicine, UNITED STATES

## Abstract

*Candida albicans* is a leading cause of intravascular catheter-related infections. The capacity for biofilm formation has been proposed to contribute to the persistence of this fungal pathogen on catheter surfaces. While efforts have been devoted to identifying microbial factors that modulate *C*. *albicans* biofilm formation *in vitro*, our understanding of the host factors that may shape *C*. *albicans* persistence in intravascular catheters is lacking. Here, we used multiphoton microscopy to characterize biofilms in intravascular catheters removed from candidiasis patients. We demonstrated that, NETosis, a type of neutrophil cell death with antimicrobial activity, was implicated in the interaction of immune cells with *C*. *albicans* in the catheters. The catheter isolates exhibited reduced filamentation and candidalysin gene expression, specifically in the total parenteral nutrition culture environment. Furthermore, we showed that the ablation of candidalysin expression in *C*. *albicans* reduced NETosis and conferred resistance to neutrophil-mediated fungal biofilm elimination. Our findings illustrate the role of neutrophil NETosis in modulating *C*. *albicans* biofilm persistence in an intravascular catheter, highlighting that *C*. *albicans* can benefit from reduced virulence expression to promote its persistence in an intravascular catheter.

## Author summary

Intravascular catheters are widely used to administer medication and provide nutritional support to patients. However, their clinical use also increases the incidence of bloodstream infections and constitutes a risk factor for *Candida albicans* infection. Microbial biofilms have been proposed as the primary living style of microbes on a surface, including those on intravascular catheters. Numerous studies focus on identifying microbial factors contributing to *C*. *albicans* biofilm formation. However, our understanding of the host factors involved in controlling *C*. *albicans* biofilms on the surface of intravascular catheters is limited. In this study, we demonstrated that the biofilms in intravascular catheters removed from candidiasis patients comprised not only *C*. *albicans* cells but also human immune cells. Among these catheters, the immune cells interacting with the fungus exhibited different properties of NETosis, a type of neutrophil cell death with antimicrobial activity. We further showed that the catheter isolates exhibited reduced expression of candidalysin. Supplementation of candidalysin promoted NETosis induced by *C*. *albicans* biofilms and diminished *C*. *albicans* biofilms on a surface. Our findings highlight the implication of neutrophils in eliminating *C*. *albicans* biofilms, and point out the benefit of reduced candidalysin expression for *C*. *albicans* to persist in intravascular catheters.

## Introduction

Invasive candidiasis, including the bloodstream infection called candidemia, is infection of *Candida* species at sterile sites in human body [1]. *Candida albicans* accounts for more than half of candidiasis with the mortality rate of up to 40%. Invasive candidiasis is highly associated with the use of intravascular catheters, where the catheters are considered to be colonized by *C*. *albicans* as biofilms before the microbes disseminate into the blood.

Microbial biofilms are communities of aggregated microbial cells. Biofilms are the predominant growth state on a surface of many microorganisms, including environmental microbes and clinically important pathogens [2–4]. *C*. *albicans* biofilms can be found on the intravascular catheters from patients and the mucosal surfaces *in vivo* in mouse models [5–7]. Once it forms on a intravascular catheter, *C*. *albicans* cells within the biofilms can potentially disseminate to bloodstream to cause systemic candidiasis and deep-seated tissue infection [1,5]. High intravascular antifungal doses together with removal of the contaminated catheters are generally required to treat the infections, even though this management can cause serious complications and are costly [8–10]. Biofilm formation capacity of *C*. *albicans* is thus suggested to be critical for candidiasis, especially in patients with intravascular catheters [1,4].

Catheters are medical devices implanted into patients for therapeutic purposes. A central venous catheter is a tube placed into a large vein to give medications to patients. Patients who have central venous catheters often receive medications, such as antimicrobials, immunosuppressant, and total parenteral nutrition (TPN) via the catheters [11,12]. These medications can modulate the physiology of the fungi and immune cells and therefore the consequence of the interactions between fungi and immune cells in blood and catheters [12–17].

*C*. *albicans* is a dimorphic fungus capable of thriving a wide range of extreme environments [18]. The morphological transition takes place at 37°C and confers biofilm formation. The yeast-to-hypha filamentation process is required for *C*. *albicans* full virulence. It has been demonstrated that expression of *C*. *albicans* virulence genes, such as *ECE1*, is associated with filamentation and finely regulated by transcription factors controlling morphological transition [19,20]. Accordingly, *C*. *albicans* locked in either yeast or filamentous form due to ablation of the transcription factors is less virulent and may show decreased biofilm formation capacity than the parental strain [20–23].

Various *in vitro* models have been established to recapitulate *C*. *albicans* biofilm formation on an intravascular catheter in clinical infections, mainly focusing on the use of relevant plastic materials for microbial adhesion [24–28]. *C*. *albicans* biofilm formation is generally thought to comprise four stages: (i) adherence of spherical yeast cells to a surface, (ii) initiation of cell proliferation to form a basal layer of anchoring cells, (iii) formation of filamentous structure concomitant with extracellular matrix production, and (iv) dispersal of yeast-form cells from the biofilm mass to seed a new site [4]. Multiple transcriptional regulators were identified in *C*. *albicans*, mostly in the reference strain SC5314, to be critical for biofilm formation [29]. Non-regulatory genes such as *ALS* family genes and *HWP*, which are highly expressed in filamentation growth, are also involved in biofilm formation [4]. However, these studies have overlooked potential biodiversity of *C*. *albicans*, in which biofilm formation capacity has been demonstrated to be heterogeneous among the clinical isolates [30–32].

While biofilm formation has been proposed to be critical for clinical isolates to achieve successful colonization and infection, clinical isolates appeared to exhibit lower biofilm formation capacity as compared to several domestic reference strains derived from SC5314, which was isolated before the 1980s [23]. The high biofilm formation capacity of SC5314 can be partly attributed to a gain-of-function mutation in *ROB1*, which has not been found in *C*. *albicans* clinical isolates [23]. This suggests that the evolution of *C*. *albicans* may be shaped by additional factors that would not result in selection of the gain-of-function mutation in *ROB1*.

In this study, we aimed to identify the host factors shaping *C*. *albicans* biofilm formation in intravascular catheters. We characterized the biofilms on the intravascular catheters removed from candidiasis patients with multi-photon microscopy. We detected infiltration of immune cells associated with fungal cells at the inner surfaces of the catheters. In some but not all the catheters, we noticed the presence of neutrophil extracellular traps (NETs), NET-like structures (NLS), and cells undergoing NETosis, a type of neutrophil cell death resulting in release of NETs and NLS. We further characterized the *C*. *albicans* isolates from the catheters under TPN environment, and showed that these clinical isolates exhibit low expression of candidalysin. The isolates that can resist neutrophil-mediated biofilm removal under TPN environment *in vitro* also showed undetectable NETosis in the corresponding intravascular catheters. Whereas, supplementation of candidalysin induced NETosis and facilitated the removal of *C*. *albicans* biofilms. Our findings reveal the importance of candidalysin downregulation in *C*. *albicans* persistence in intravascular catheters.

## Results

### Detection of fungal biofilms at the inner surfaces of the intravascular catheters from candidiasis patients

**Fig 1:**
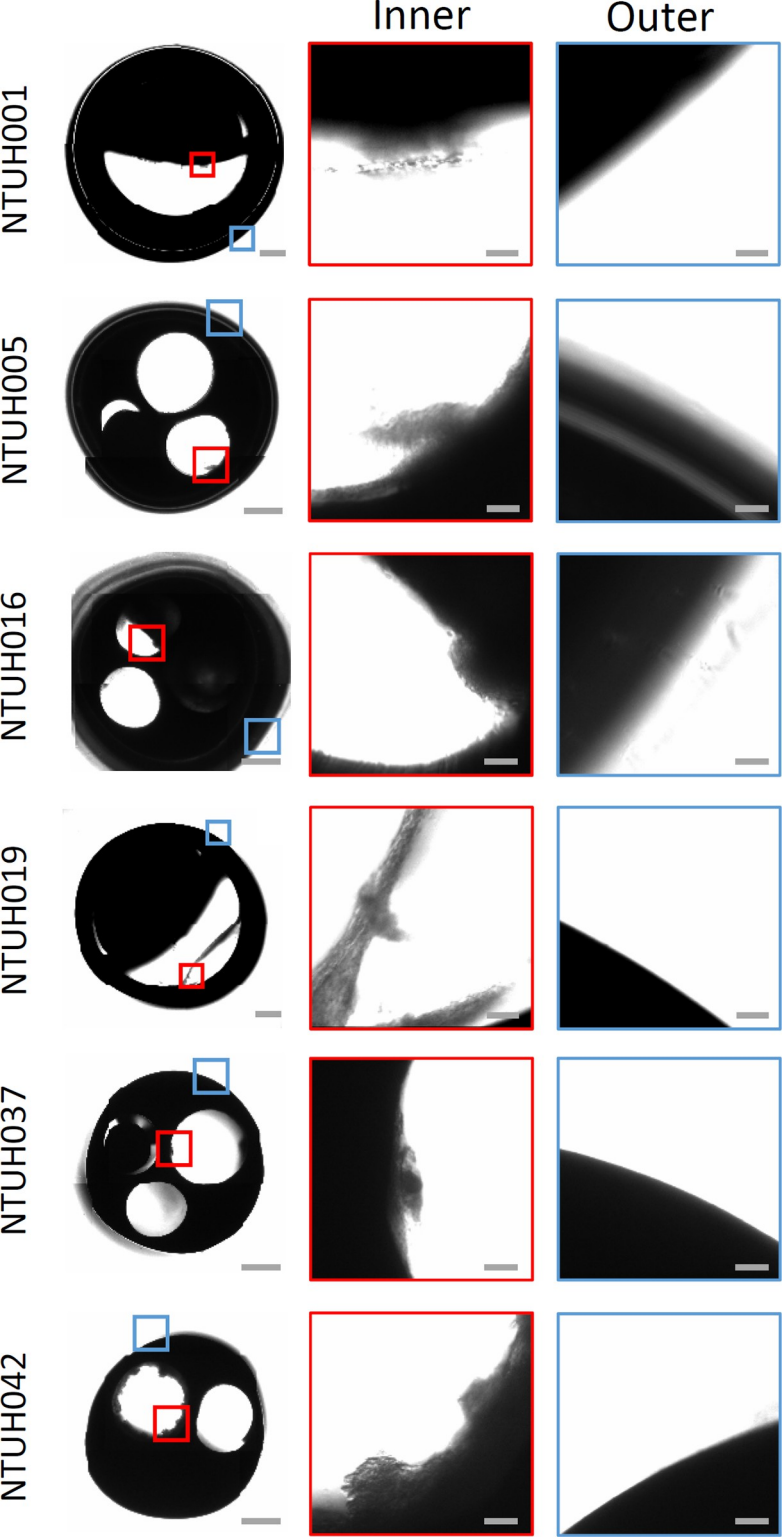
Identification of biofilms at the inner surfaces of the catheters removed from candidemia patients. The catheters were taken for cross-section imaging by bright field microscopy. Magnified images of the inner and outer surfaces of the catheters were shown in red and blue boxes as indicated. Scale bar, 500 μm for whole catheters, and 20 μm for magnified inner and outer surfaces.

We collected nine intravascular catheters removed from candidiasis patients where the catheters were also reported to be positive of *C*. *albicans* culture. A high fungal load (> 10 CFUs) in tip culture was found in six of the catheters. These patients did not receive any antifungal within 3 days before catheter removal (Table 1). We investigated the colonization sites of *C*. *albicans* by imaging the cross sections of the catheters for any unusual cell aggregation, which would potentially be fungal biofilms (Fig 1). While the outer surfaces of the intravascular catheters were completely smooth without any possible biofilms, aggregation of cells was noted at the inner luminal surfaces of all the six catheters (Fig 1).

**Table 1:** Clinical summary of the patients.

Patient No.	Age	Gender	Catheter type	TPN	Antibiotics	Antifungal	Malignancy	Chemotherapy	WBC (No./μL)	Diagnosis	Outcome
NTUH001	87	M	DLC	N	Meropenem	N	Prostate cancer	N	10770	Bacteremia	D
NTUH005	73	M	CVC	Y	Ceftazidime	N	Rectal carcinoma	FOLFOX	17700	Fungemia	D
NTUH016	74	M	CVC	Y	Piperacillin/tazobactam	N	Rectal carcinoma	Xeloda	9760	Fungemia	S
NTUH019	38	M	DLC	N	Ceftazidime/colistin	N	N	N	21000	Fungemia	D
NTUH037	85	F	CVC	N	Cefepime	N	N	N	13900	Paraspinal abscess	S
NTUH042	90	M	CVC	Y	Ceftazidime/avibactam + meropenem	N	N	N	7210	Urosepsis	S

M, male. F, female. DLC, double lumen catheter. CVC, central venous catheter. Y, yes. N, no treatment. WBC, white blood cells. D, died. S, survived.

**Fig 2:**
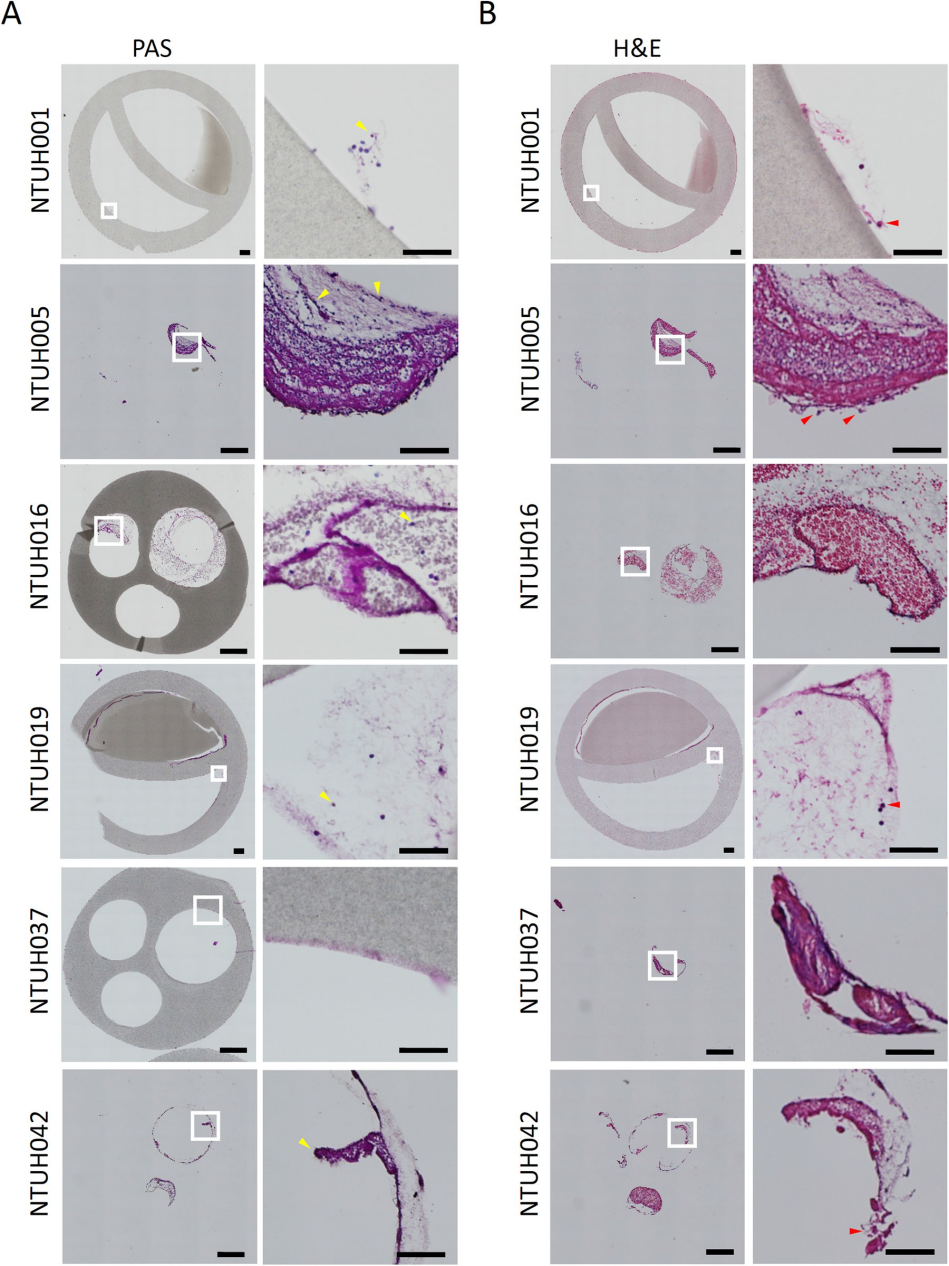
Detection of fungal cells at the inner surfaces of the catheters. The catheters were fixed with paraformaldehyde followed by paraffin embedding and sectioning to 10 μm thickness. (A) Periodic acid-Schiff (PAS) staining was performed to detect fungal cells in the catheters. Fungal cells are indicated by yellow arrows. (B) Hematoxylin and eosin staining (H&E) was applied for detection of human cell components. Polymorphonuclear cells associated with a fibrous structure are indicated by red arrows. Scale bar, 500 μm for whole catheters, and 50 μm for magnified images at the inner surfaces of the catheters.

To validate the presence of fungal cells within the cell aggregates at the inner surface, we stained the sections of the intravascular catheters by Periodic acid-Schiff (PAS) staining (Fig 2A). The PAS^+^ fungal cells appeared to be filamentous and associated with a fibrous structure in the catheters removed from patients NTUH001 and NTUH005. Whereas, the fungal cells in the intravascular catheters of patients NTUH016 and NTUH019 showed yeast morphology, and those of patient NTUH042 exhibited aggregation (Fig 2A). Hematoxylin and eosin staining (H&E) was performed to address potential infiltration of immune cells in the intravascular catheters (Fig 2B). We observed the infiltration of polymorphonuclear cells (PMNs) associated with the fibrous structure in the lumen of the intravascular catheters, which were highly abundant in the catheter of NTUH005 and to a lesser content in that of NTUH001 (Fig 2B). Briefly, these data show the presence of biofilms, comprising both fungal cells and immune cells specifically at the luminal surfaces of the intravascular catheters removed from candidiasis patients.

### The *Candida*-associated immune cells in the catheters displayed diverse phenotypes

**Fig 3:**
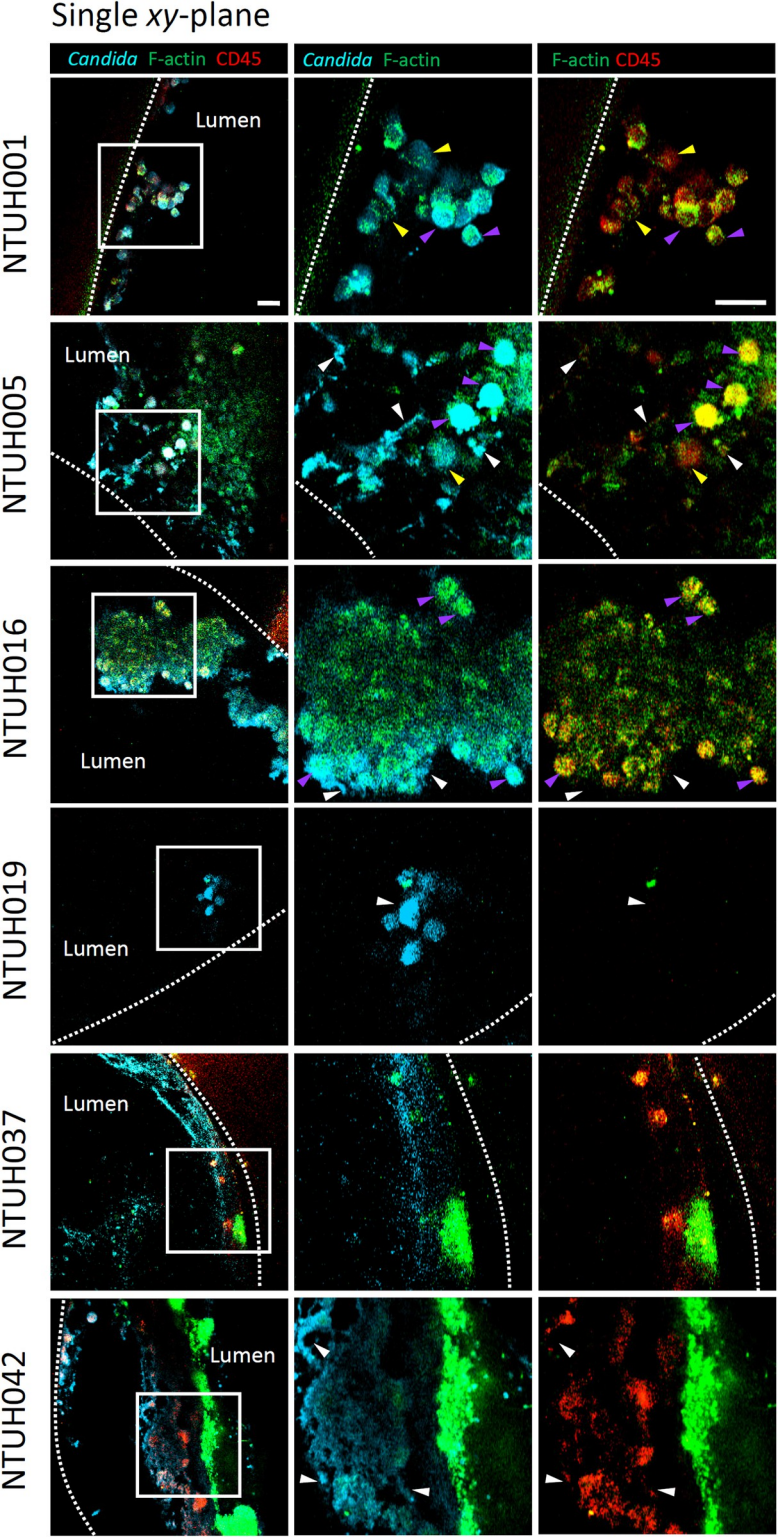
Infiltration of human immune cells and their association with *Candida* cells at the inner surfaces of the catheters. Whole mount catheters (2 mm in length) were fixed and stained for the presence of *Candida* cells and human immune cells, followed by observation under a two-photon microscope. Single *xy*-plane images are shown. *Candida* cells and human CD45^+^ F-actin^+^ immune cells associated with *Candida* antigens are indicated by white and magenta arrows, respectively. CD45^+^ F-actin^-^ immune cells showing actin cytoskeleton disassembly are indicated by yellow arrows. Scale bar, 20 μm.

**Fig 4:**
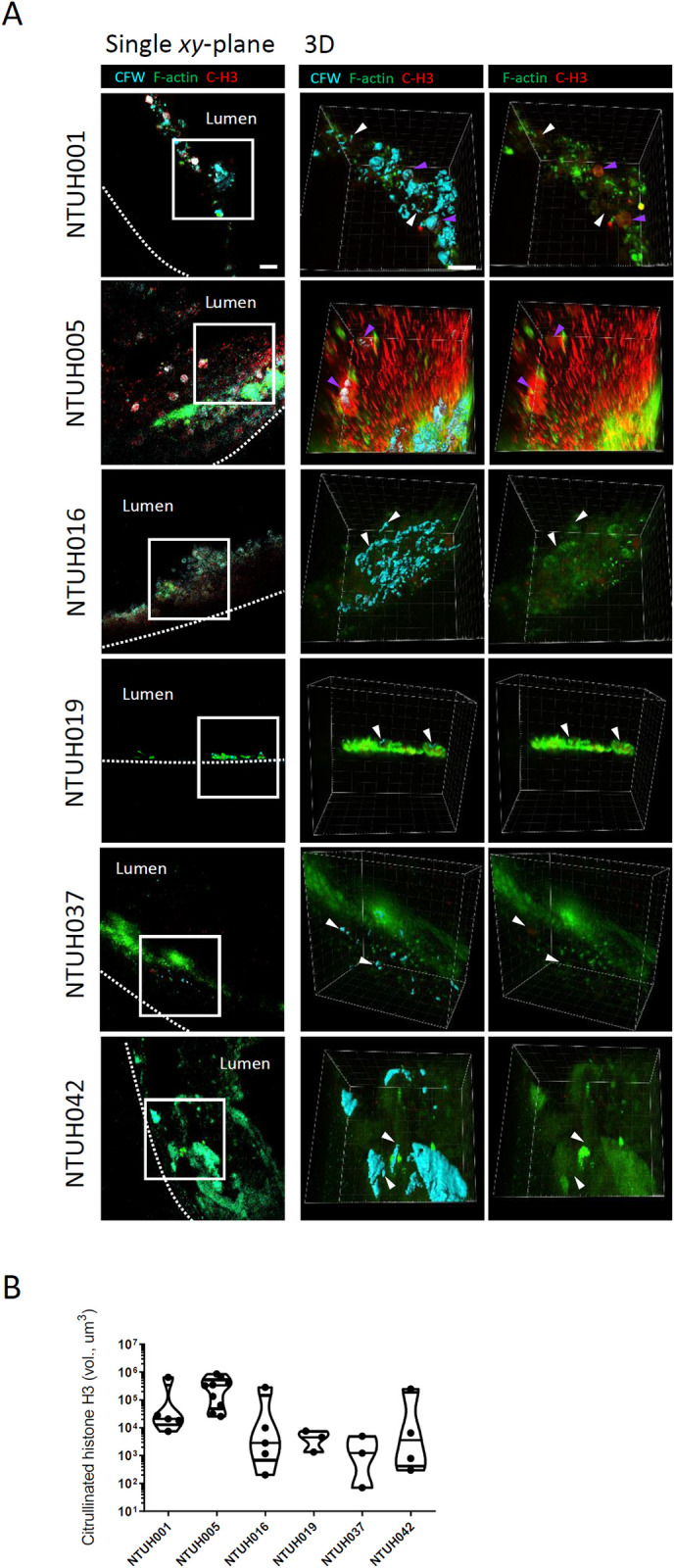
Neutrophil extracellular traps were associated with *Candida* cells in the catheters. The catheters were processed as described in Fig 3. Antibodies against citrullinated histone H3 (C-H3) were used to detect neutrophil extracellular traps (NETs). *Candida* cells were stained by Calcofluor white. Immune cells were identified by F-actin staining with phalloidin. (A) Single *xy*-plane and reconstituted 3D (z = 60 μm) images are shown. *Candida* cells associated with NETs are indicated by magenta arrows, while *Candida* cells free from NETs are indicated by white arrows. Scale bar, 20 μm. (B) Violin plot for comparison of NETs quantified by the volume of C-H3 signals in the stacked images obtained from the catheters. n = 3 to 9. Each point represents one stacked image.

To validate the presence of *C*. *albicans* and human immune cells in the cell aggregation observed at the inner surfaces of the catheters, we imaged the cross-section view of the whole catheter by two-photon microscopy to preserve the structure of the biofilms associated to the catheters. We demonstrated that the biomass at the luminal surfaces of the catheters was recognized by an anti-*Candida* antibody (Fig 3) as well as calcofluor white (Fig 4A), both of which stain *C*. *albicans* cells, suggesting the presence of *Candida* cells there. In addition to the fungal cells showing yeast or filamentous morphology were recognized by the anti-*Candida* antibody (Fig 3, white arrows), we found that some infiltrating CD45^+^ F-actin^+^ immune cells, especially those in the intravascular catheters of NTUH001 and NTUH005, also showed positive of *Candida* staining (Fig 3, magenta arrows). This was possibly due to internalization of *Candida* antigens by these cells or association of NLS released by NETotic cells with damaged fungal cells. We also observed the presence of CD45^+^ F-actin^-^ cells showing actin cytoskeleton disassembly, a hallmark of early NETosis of neutrophils, associated with *Candida* antigens in the clusters (Fig 3, yellow arrows) [33,34]. Of note, these CD45^+^ F-actin^-^ cells were not associated with *Candida* cells in the catheters of NTUH016, NTUH019, NTUH037 and NTUH042 (Fig 3). The lack of CD45^+^ F-actin^-^ cells in these catheters was not due to lower number of white blood cells (WBC) in these patients as all the patients showed comparable counts of WBC in the blood (Table 1).

The presence of CD45^+^ F-actin^-^ cells led us to investigate the existence of NETosis in the intravascular catheters using an antibody recognizing citrullinated histone H3 (citH3), which is a marker for NETotic cells, NLS and NETs (Fig 4) [35]. We demonstrated that in the catheter of NTUH005 there was a high abundance of citH3^+^ NLS capturing fungal cells (Fig 4A, NTUH005, magenta arrows). We also detected citH3^+^ F-actin^-^ cells, which represent NETotic cells, associated with fungal cells in the catheter of NTUH001 (Fig 4A, NTUH001, magenta arrows). In contrast, neither the citH3^+^ NLS nor the citH3^+^ F-actin^-^ cells were seen in the other intravascular catheters (Fig 4A, NTUH016, NTUH019, NTUH037 and NTUH042). Quantification of citH3 volume in the catheters indicates that citH3^+^ NETotic cells, NLS and NETs were rare in the catheters of NTUH016, NTUH019, NTUH037 and NTUH042, but highly abundant in that of NTUH005 (Fig 4B). Of note, while filamentous structure has been demonstrated to be the major form of *C*. *albicans* biofilms, we did not observe a cluster of filamentous fungal cells in the catheters, and most of the fungal cells displayed short filament and yeast morphology in the intravascular catheters (Figs 3 and 4A). These findings show that the biofilms on the intravascular catheters contain infiltrating immune cells, which may undergo NETosis to exert antifungal activity.

### The catheter isolates exhibit distinct filamentation capacity and virulence gene expression compared to the reference strain SC5314

**Fig 5:**
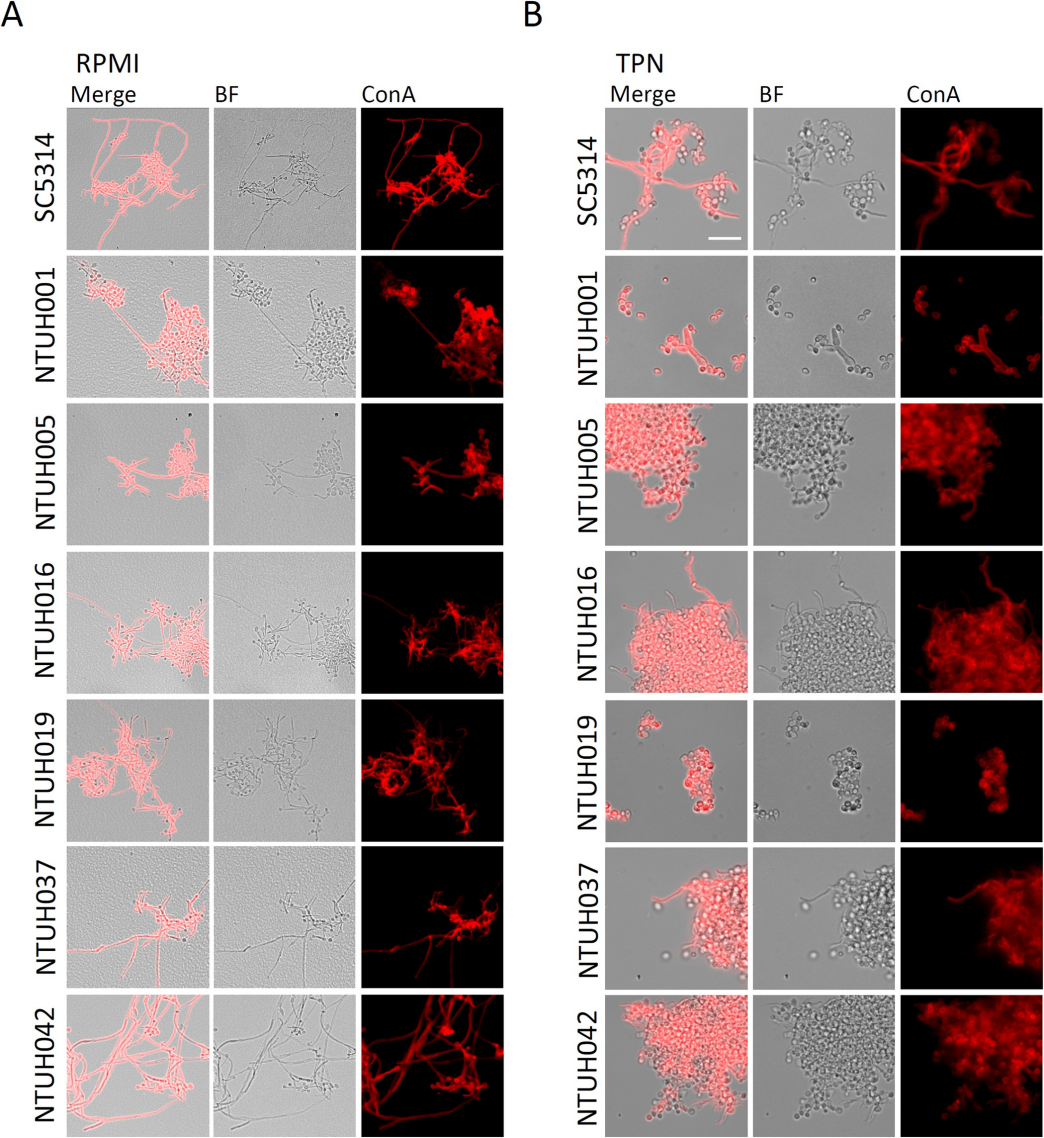
Morphology of the catheter isolates cultured in RPMI or TPN. *C*. *albicans* cells were cultured in RPMI medium (A) or TPN (B) for 24 hours at 37°C, 180 rpm. The cells were fixed by 4% paraformaldehyde followed by Concanavalin A (Con A) staining to detect mannan. Represented images are shown. BF, bright field. Scale bar: 20 μm.

Filamentous fungal cells have been shown to be the primary form of *C*. *albicans* in biofilms and infected tissues [4]. However, we found a paucity of filamentous structure of *C*. *albicans* in the catheters (Figs 3 and 4A). It has been demonstrated that a natural clinical variant of *C*. *albicans* that cannot produce filaments displayed poor phagocytosis by neutrophils, and relatively low ROS and NETs accumulation, thereby resisting neutrophil-mediated killing [36]. We thus tested filamentation capacity of the catheter isolates (Fig 5). We incubated the catheter isolates in complete RPMI 1640 medium containing 10% serum (RPMI), a culture environment that has been demonstrated to promote *Candida* filamentation and biofilm formation [37]. All the catheter isolates displayed filamentation capacity that is comparable to the reference strain SC5314 in RPMI in planktonic culture (Fig 5A). The filamentous cells can also be observed in biofilms of all the catheter isolates cultured in RPMI, but less abundant in those of NTUH001 and NTUH005 isolates (S2 Fig, *Candida*). Briefly, all the catheter isolates possess filamentation capacity in the filamentation inducing environment, but the adhesion capacity of the filamentous cells may vary among the isolates.

The presence of biofilms specifically at the luminal surfaces of the catheters but not at the outer surfaces suggests that the environment in the catheter lumen is an important factor modulating fungal cell persistence on the catheter. TPN infusion is highly associated with candidiasis, can be found in more than 90% of candidemia patients [38,39]. Accordingly, 3 out of the 6 patients in this study received TPN at the time of candidiasis (Table 1). We thus tested filamentation capacity of the catheter isolates in TPN (Fig 5B). We found that all the catheter isolates showed reduced filamentation in TPN as compared to those in RPMI, and mainly presented yeasts or pseudohyphae in aggregates when cultured in TPN (Fig 5B). Whereas, the reference strain SC5314 preserved filamentation capacity forming true hyphae in TPN (Fig 5B). These data suggest that components in TPN may suppress *C*. *albicans* filamentation, especially in the catheter isolates.

**Fig 6:**
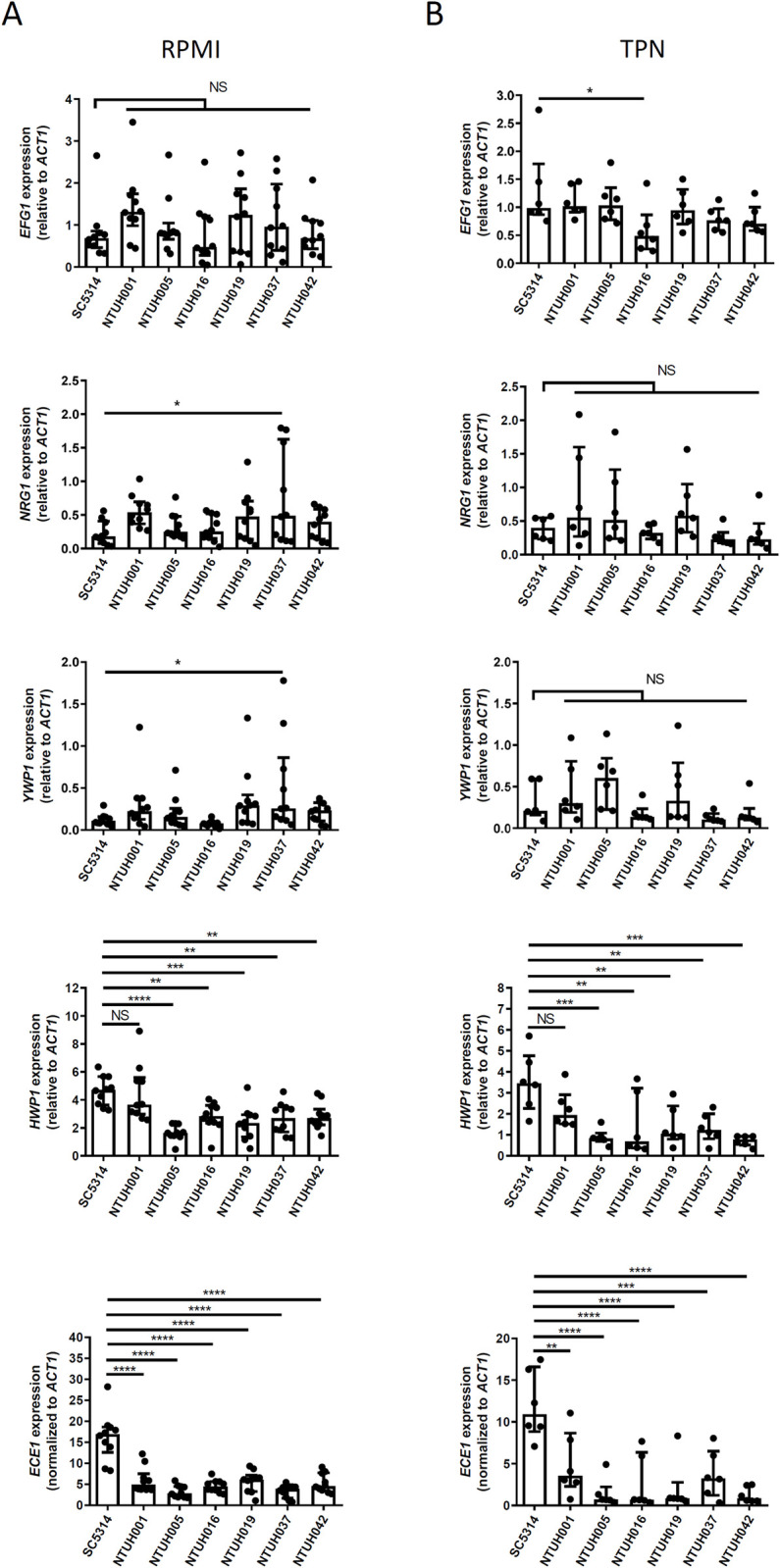
The *C*. *albicans* catheter isolates exhibited reduced expression of specific filamentation-associated genes. *C*. *albicans* yeast cells were cultured in RPMI (A) or TPN (B) at 37°C for 24 hours. Gene expression was quantified by qRT-PCR and normalized to *ACT1* expression. Data represent mean with SD from three independent experiments. Statistical analysis was performed with the one-way ANOVA, followed by Dunnett’s multiple comparison test as compared to the reference strain SC5314. *, P<0.05. **, P<0.01. ***, P<0.001. ****, P<0.0001. NS, no significant difference.

*C*. *albicans* filamentous morphogenesis is intricately regulated by several transcription factors. Nrg1 functions as a core negative regulator suppressing filamentation, while Efg1 is a negative regulator of Nrg1, thereby inducing filamentation [40]. We hypothesized that the antagonistic regulation between Efg1 and Nrg1 may underlie the morphogenesis regulation specifically in TPN. Unexpectedly, all the catheter isolates showed comparable expression of *EFG1* and *NRG1* to SC5314 in RPMI and in TPN (Fig 6A and 6B, *EFG1* and *NRG1*). Notably, relative expression of these genes to *ACT1* was also comparable between RPMI and TPN in all the strains (Fig 6A and 6B, *EFG1* and *NRG1*). This suggests that the filamentation modulation by TPN is downstream of *EFG1*-*NRG1* regulation. We also showed that expression of *YWP1*, a yeast-associated gene, was comparable among all the strains in both culture environments (Fig 6A and 6B, *YWP1*). Expression of filamentation-associated genes were also addressed. Except for NTUH001 isolate, we found that the other catheter isolates showed significantly reduced expression of *HWP1* in both RPMI and TPN cultures (Fig 6A and 6B, *HWP1*). We also studied the expression of *ECE1*, which encodes a NETosis-inducing fungal toxin called candidalysin highly expressed by hyphal filaments [41]. We demonstrated that all the catheter isolates showed an approximately 3-fold reduction in *ECE1* expression compared to that of SC5314 in RPMI, and the reduction was more than 10-fold in TPN (Fig 6A and 6B, *ECE1*). Nevertheless, expression of *ALS3* and *HGC1*, the filamentation-associated genes whose expression are less varied among clinical isolates of *C*. *albicans* [42], was comparable among the catheter isolates and SC5314 in both RPMI and TPN culture environments (S1A and S1B Fig). These data demonstrate that the catheter isolates showed a reduction of specific filamentation-associated genes, such as *ECE1*, especially when cultured in TPN.

### The interaction of neutrophils with the biofilms of the catheter isolates varied

**Fig 7:**
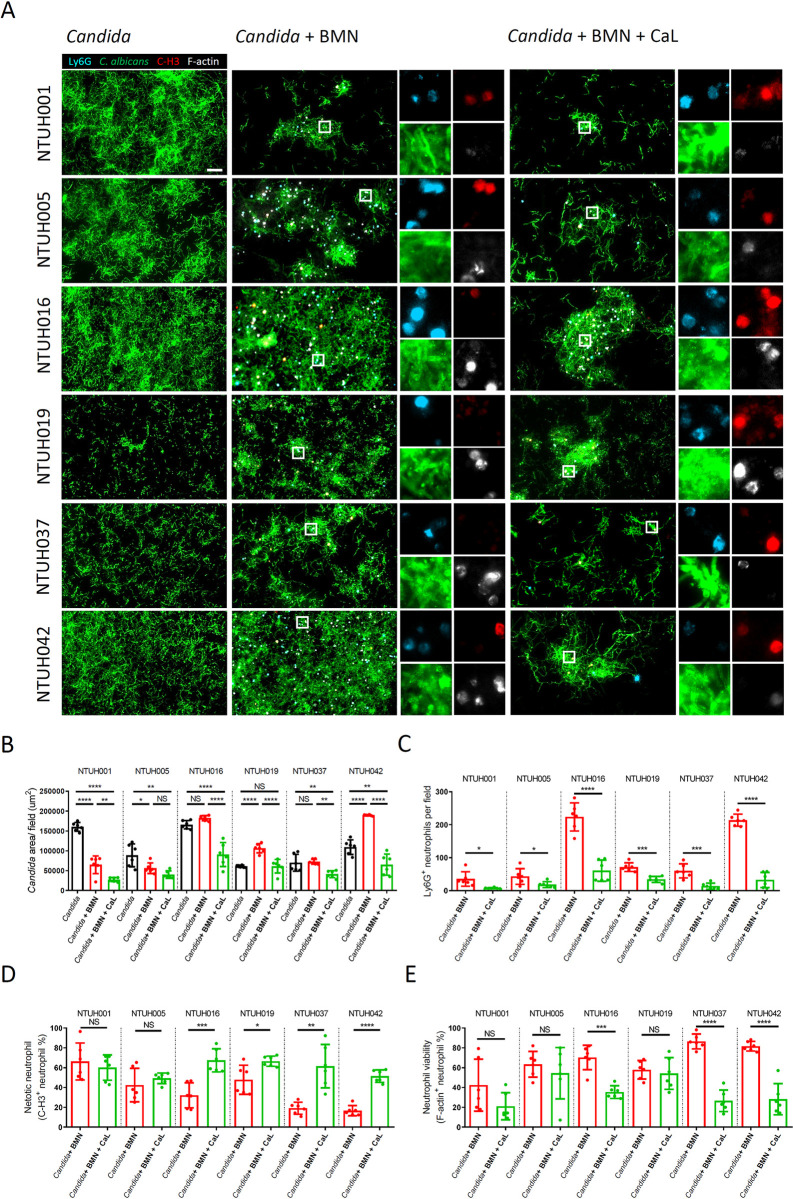
The catheter isolates exhibited diverse capacity in resistance to neutrophil-mediated fungal biofilm removal. *C*. *albicans* cells were cultured in TPN for 24 hours to allow biofilm formation on a coverslip. The biofilms developed in TPN were then incubated with mouse bone marrow-derived neutrophils (BMN) in TPN in the absence or presence of candidalysin (CaL, 15 μM) for another 6 hours before fixation and staining. BMN NETosis was observed by Ly6G (cyan), F-actin (white) and citrullinated histone H3 staining (C-H3, red). *Candida* cells were stained by mDectin-1-Fc (green) that recognizes β-glucan on fungal cell wall. (A) Representative images are shown. (B) *Candida* biofilms were quantified as the area of *C*. *albicans* cells in each field on the coverslips. (C) Infiltration of BMNs (Ly6G^+^) into the surface-associated biofilms of each isolate is shown. (D and E) The percentage of NETotic BMNs (C-H3^+^ Ly6G^+^ cells/ total Ly6G^+^ cells) (D) and viable non- NETotic (F-actin^+^ C-H3^-^ Ly6G^+^ cells/ total Ly6G^+^ cells) (E) in the biofilms is shown. All the images were analyzed with Imaris (Bitplane). (B-E) Results shown are mean with SD from 3 independent experiments, 2 images from each coverslip. Statistical analysis was performed with the one-way ANOVA, followed by Tukey’s multiple comparison test (B) or a two-tailed unpaired Student *T* test (C-E). *, P<0.05. **, P<0.01. ***, P<0.001. ****, P<0.0001. NS, no significant difference. Scale bar, 50 μm.

The distinct biofilm structure and immune cell properties in the intravascular catheters led us to investigate the biofilm structure of the catheter isolates, and the interaction of the biofilms with immune cells (Figs 2–4). The biofilms of these isolates displayed almost single-layer structure in TPN (Fig 7A, *Candida*). Biofilm formation capacity in TPN varied among the clinical isolates, where the isolates of NTUH019 and NTUH037 showed the lowest biofilm area and reduced filamentation in the biofilms (Fig 7A and 7B, *Candida*). We then studied the interaction between *C*. *albicans* biofilms and mouse bone marrow derived neutrophils (BMN) in TPN. While further incubation with mouse BMN reduced the biofilms of NTUH001 and NTUH005 isolates, these immune cells did not modulate the biofilms of NTUH016 and NTUH037 isolates, but even increased the biofilms of NTUH019 and NTUH042 isolates (Fig 7A and 7B, *Candida* and *Candida* + BMN). The biofilms of NTUH001 and NTUH005 isolates did not contain more neutrophils, suggesting that the ability of the other isolates to resist BMN-mediated biofilm elimination was not due to decreased neutrophil infiltration to the biofilms of these isolates (Fig 7A–7C, *Candida* + BMN).

Due to the low expression of *ECE1* in the clinical isolates, we hypothesized that candidalysin-induced NETosis can affect *C*. *albicans* biofilm persistence modulated by neutrophils [41]. Accordingly, we were able to detect NETotic cells expressing Ly6G and citH3 with deformed F-actin cytoskeleton, which were relatively fewer in the biofilms of NTUH037 and NTUH042 isolates in TPN (Fig 7A and 7D, *Candida* + BMN). The lower NETosis induction property of NTUH037 and NTUH042 isolates was also in line with higher viability of neutrophils with structured F-actin cytoskeleton (Fig 7A and 7E, *Candida* + BMN). In contrast to the TPN culture, culture of BMN with the biofilms in RPMI removed the biofilms of most of the catheter isolates as well as that of SC5314 (S2A and S2B Fig).

To address the potential involvement of low *ECE1* expression of the catheter isolates in NETosis induction and resistance to BMN-mediated fungal biofilm elimination, we treated candidalysin peptides (Ece1-III_62-93_) together with BMN to *C*. *albicans* biofilms in TPN environment. Candidalysin supplementation significantly reduced the biofilms of almost all the catheter isolates except for that of NTUH005 isolate (Fig 7A and 7B). The treatment largely reduced the number of BMN associated with the biofilms possibly due to the shedding of biofilms together with NETotic BMN (Fig 7C). In contrast, candidalysin treatment had no effect on NETosis in the biofilms of NTUH001 and NTUH005 isolates, which showed more NETotic BMN in the absence of candidalysin (Fig 7A and 7D, NTUH001 and NTUH005). Whereas, candidalysin promoted NETosis and decreased F-actin^+^ BMN when incubated with the biofilms of the other catheter isolates (Fig 7A, 7D and 7E, NTUH016, NTUH019, NTUH037 and NTUH042). Candidalysin treatment in RPMI did not further induce NETosis when incubated with *C*. *albicans* biofilms, but even decreased the number of NETotic cells in the biofilms of NTUH001 isolate (S2C Fig). This suggests that the effect of candidalysin supplementation on NETosis in *C*. *albicans* biofilms was specific to TPN environment. Briefly, the catheter isolates from NTUH016, NTUH019, NTUH037 and NTUH042 can resist BMN-mediated biofilm removal specifically in TPN environment. The resistance can be overcome by candidalysin supplementation which triggers NETosis in the biofilms.

### Disruption of candidalysin expression reduced NETosis of biofilm infiltrating neutrophils and enhanced *C*. *albicans* biofilm persistence in the presence of neutrophils

**Fig 8:**
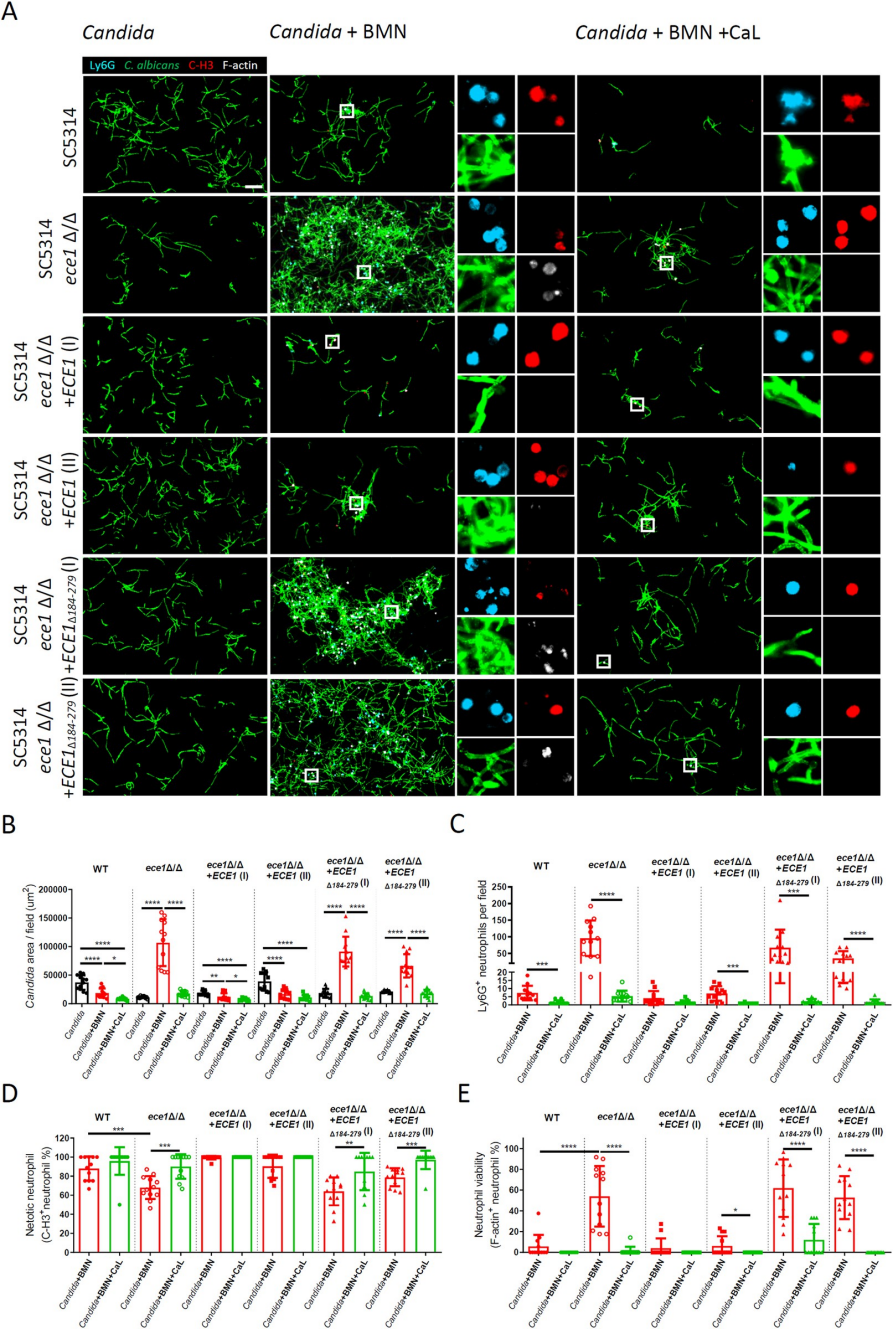
Candidalysin induced NETosis of biofilm-infiltrating neutrophils and removal of *Candida* biofilms. Bofilm formation assay of the reference strain SC5314, the candidalysin deficient isogenic mutants (*ece1*Δ/Δ, *ece1*Δ/Δ+*ECE1*_Δ184–279_ [I] and *ece1*Δ/Δ+*ECE1*_Δ184–279_ [II]), and the *ECE1* revertant strains (*ece1*Δ/Δ+*ECE1* [I] and *ece1*Δ/Δ+*ECE1* [II]) was performed as described in Fig 7. (A) Representative images are shown. (B) *Candida* biofilms were quantified as the area of *C*. *albicans* cells (Fc-mDectin-1^+^) in each field on the coverslips. (C) Infiltration of BMNs (Ly6G^+^) into the surface-associated biofilms of each isolate is shown. (D and E) The percentage of NETotic BMNs (C-H3^+^ Ly6G^+^ cells/ total Ly6G^+^ cells) (D) and viable non- NETotic (F-actin^+^ C-H3^-^ Ly6G^+^ cells/ total Ly6G^+^ cells) (E) in the biofilms is shown. All the images were analyzed with Imaris (Bitplane). (B-E) Results shown are mean with SD from 3 independent experiments, 2 images from each coverslip. Statistical analysis was performed with the one-way ANOVA, followed by Tukey’s multiple comparison test (B) or a two-tailed unpaired Student *T* test (C-E). **, P<0.01. ***, P<0.001. ****, P<0.0001. NS, no significant difference. Scale bar, 50 μm.

**Fig 9:**
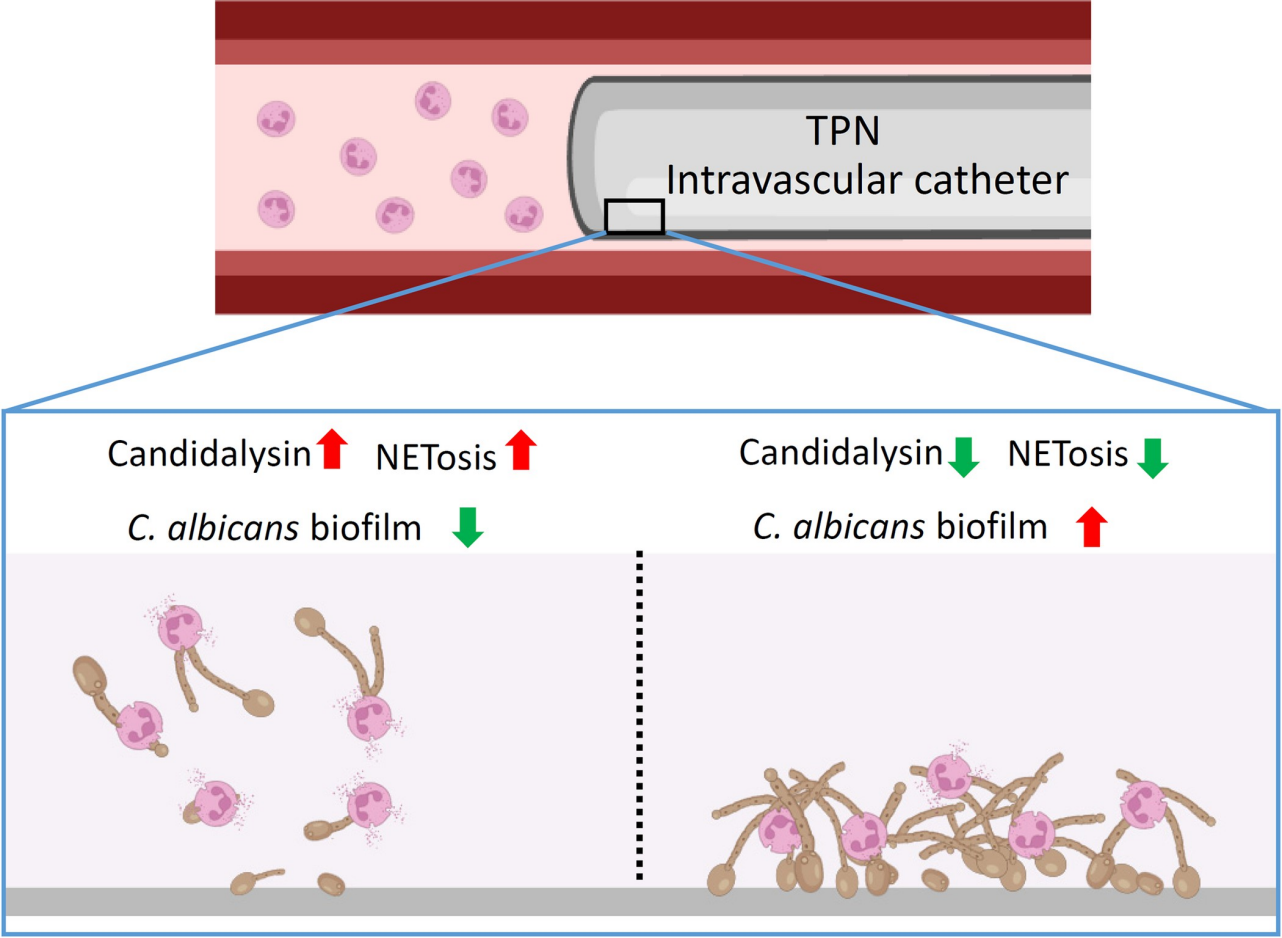
A model for the function of candidalysin in inducing NETosis and *C*. *albicans* biofilm elimination in an intravascular catheter. The biofilms of *C*. *albicans* clinical isolates exhibited different ability in promoting NETosis. Supplementation of candidalysin induced NETosis and facilitated *C*. *albicans* biofilm elimination Whereas, ablation of candidalysin expression in the high candidalysin-producing strain reduced NETosis and increased *C*. *albicans* persistence on a surface. Created with Biorender.com.

We specifically investigated the effect of candidalysin on NETosis and *C*. *albicans* biofilm persistence on a surface in TPN environment using the reference strain SC5314 and its isogenic candidalysin deficient mutants (*ece1*Δ/Δ, *ece1*Δ/Δ+*ECE1*_Δ184–279_ [I] and *ece1*Δ/Δ+*ECE1*_Δ184–279_ [II]) (Fig 8). We demonstrated that BMN was able to remove the biofilm of the wild-type SC5314, but increased that of the three candidalysin deficient mutants (Fig 8A and 8B). There were more Ly6G^+^ BMN infiltrating into the biofilm of the candidalysin deficient mutants, but the percentage of NETotic BMN was lower accompanying by more F-actin^+^ viable BMN in the biofilm of the candidalysin deficient mutants, compared to that of SC5314 (Fig 8A and 8C). Whereas, the two *ECE1* revertant strains (*ece1*Δ/Δ+*ECE1* [I] and *ece1*Δ/Δ+*ECE1* [II]) showed comparable biofilms and NETotic induction activity to that of SC5314 (Fig 8). Treatment of exogenous candidalysin peptides to the *C*. *albicans* biofilms with BMN promoted BMN NETosis, decreased F-actin^+^ BMN, and abolished the resistance of all three candidalysin deficient mutants to BMN-mediated biofilm elimination (Fig 8). Together, expression of candidalysin by *C*. *albicans* induced NETosis and decreased persistence of *C*. *albicans* biofilm in the presence of neutrophils (Fig 9).

## Discussion

*C*. *albicans* is one of the main pathogens causing intravascular catheter-related infections possibly due to its high biofilm formation capacity on the catheters [38]. In the present study, we characterized the biofilms on the intravascular catheters of candidiasis patients using multi-photon microscopy. We demonstrated the implication of NETosis in the interaction between *C*. *albicans* cells and patient immune system at the luminal surfaces of the catheters, suggesting a role of NETosis in shaping *C*. *albicans* colonization in an intravascular catheter. Accordingly, components of NETs, such as histone proteins and myeloperoxidase were found to be associated with *C*. *albicans* biofilms in a rat venous catheter infection model [43]. Using the *in vitro* biofilm formation model, we further showed that candidalysin can promote NETosis and fungal biofilm removal, highlighting that candidalysin downregulation by the clinical isolates enhances their surface colonization under TPN environment. Our findings may also, at least in part, explain why the gain-of-function *ROB1*^*946S*^ allele, despite increasing *in vitro* biofilm formation and virulence expression, such as candidalysin overproduction, is very rare in *C*. *albicans* clinical isolates [23].

We identified biofilms comprising both fungal cells and host immune cells only at the luminal surfaces, but not at the outer surfaces, of the intravascular catheters removed from patients. Of note, while hyphal filaments are the main cell population in diverse *in vitro* studies [4,43–45], we only found *Candida* yeast cells and short filaments in the catheter-associated biofilms. The specific location of *C*. *albicans* biofilms at the luminal surface of an intravascular catheter from a patient was also seen in another previous study [7]. We thus proposed that the different environments between the inner luminal and outer surfaces of an intravascular catheter may underlie the tropism of *C*. *albicans* biofilm on the catheters. Accordingly, we showed that in the RPMI culture mimicking a blood environment suitable for immune cell growth, *C*. *albicans* exhibited filamentous structure and the biofilms were readily eliminated by neutrophils. Whereas, in TPN environment that can be found in the lumen of an intravascular catheter, filamentation of the catheter isolates was reduced and most of the isolates can resist neutrophil-mediated biofilm removal. The reduced filamentation and expression of filamentation associated virulence genes in *C*. *albicans* could be due to the fat emulsion of TPN, since these lipids were demonstrated to downregulate expression of *HGC1* and *UME6*, both of which are positive regulators of *C*. *albicans* filamentation [14]. Of note, *UME6* is downstream of Nrg1 regulation, explaining the comparable expression of *NRG1* between RPMI and TPN environment in our finding [46]. In addition to the fat emulsion, the mild acidic environment in the TPN (pH = 6) may regulate the expression of *PHR2*, which is upregulated in acidic environment and controls *C*. *albicans* morphogenesis, cell wall structure, and virulence expression [47–49]. Accordingly, *PHR2* expression was significantly increased in SC5314 when cultured in the TPN as compared to that in RPMI (S1C Fig, SC5314). The *PHR2* upregulation in TPN culture environment was also observed in most of the catheter isolates, where the isolates of NTUH016 and NTUH042 were less responsive (S1C Fig). TPN culture environment may therefore exhibit pleiotropic effect on *C*. *albicans*, even in a strain-dependent manner. Besides, the effect of TPN environment on immune cell physiology is nevertheless unexplored and will be addressed in future studies.

Neutrophils are indispensable in controlling fungal infections. Neutrophil antimicrobial activity deficit, such as NADPH oxidase deficiency, results in impaired production of reactive oxygen species (ROS) of neutrophils and susceptibility to invasive candidiasis [50]. The ROS generated by neutrophil NADPH oxidase can induce NETosis followed by release of NETs and NLS, both of which are proinflammatory and exert antimicrobial activity [41]. We found NETs in the intravascular catheter of NTUH005 by H&E straining and two-photon microscopy with citH3 staining. However, *C*. *albicans* cells appeared to be associated to NLS with more compact structure than classical fibrous NETs induced by PMA activation [41]. The NLS was also found to be induced by filamentous *C*. *albicans* cells in a candidalysin-dependent manner [41,51]. Despite of the importance of neutrophils and NETosis in controlling *C*. *albicans* infection in humans, NETosis does not seem to be able to kill *C*. *albicans*, but to suppress or merely delay fungal growth in various *in vitro* models [16,41]. Our findings provide the evidence that NETosis can eliminate *C*. *albicans* biofilms, suggesting that neutrophil infiltration into intravascular catheters may contribute to dissociation of fungal cells, which might be captured by phagocytic cells that we could observe in the intravascular catheters removed from candidiasis patients. Future work will delineate the potential involvement of other immune cells together with neutrophils in controlling *C*. *albicans* infection in an intravascular catheter.

We observed NETosis and NLS in the catheter of NTUH001 and NTUH005. Accordingly, neutrophils specifically eliminated the biofilms formed by the corresponding isolates but not those of the other catheter isolates. The patients NTUH001 and NTUH005 did not present leukocytosis as compared to the other patients. These results suggest that the absence of NETosis and NLS in the catheters of NTUH016, NTUH019, NTUH037 and NTUH042 could be intrinsic to the property of *C*. *albicans* in the catheters. Despite of the NETosis induction capacity by NTUH001 and NTUH005 isolates, we showed that these two isolates exhibited low *ECE1* expression similar to all the catheter isolates. Moreover, supplementation of candidalysin did not promote neutrophil-mediated biofilm removal against the two isolates. These results suggest that the two catheter isolates may induce NETosis in a candidalysin-independent manner. In addition to candidalysin, extracellular matrix of *C*. *albicans* biofilms was shown to induce NETosis [41,52]. Further studies will be performed to investigate the implication of biofilm matrix and other microbial factors in inducing NETosis by NTUH001 and NTUH005 isolates.

In summary, this study shows the implication of candidalysin in modulating NETosis and *C*. *albicans* persistence on the intravascular catheters. While NETosis can assist elimination of catheter-associated *C*. *albicans* biofilm, the NETs and NLS released by NETotic cells can result in adverse consequence on the host by inducing systemic inflammatory responses [53]. Accordingly, NTUH001 and NTUH005, the two patients showed NETosis in their catheters, died from the infection. Whereas, only 1 from the rest of the 4 patients died from the infection. A systemic cohort study will be required to elucidate the impact of NETosis in intravascular catheters on the consequence of intravascular catheter-related infections.

## Materials and methods

### Ethics statement

The study with a waiver of consent for medical record collection was approved by the Institutional Review Board of National Taiwan University Hospital (202008065RINA and 202112221RIND).

### Subject

The catheter isolates were from six independent candidiasis patients with positive culture of *C*. *albicans* in the catheter culture between Aug. 2020 and Feb. 2021. The clinical information was retrospectively collected from medical records. All the patients were adults (> 18 yr old), and the clinical information is listed in Table 1.

### Microbial culture

The clinical isolates used in this study were identified and confirmed by MALDI-TOF analysis. All the *C*. *albicans* strains were routinely cultivated in YPD (1% yeast extract, 2% peptone, and 2% glucose) at 28°C with shaking at 180 rpm. All the chemicals for the routine fungal culture were procured from Bionovas Biotechnology. For characterization of *C*. *albicans* cell morphology, overnight culture of *C*. *albicans* cells at 28°C in YPD was adjusted to the OD600 = 0.5, and cultured in complete RPMI culture media (RPMI [Simply Biologics] with 10% FBS [Biological Industries]) or the total parenteral nutrition Oliclinomel N4-550E Emulsion for Infusion (Baxter) at 37°C with shaking at 180 rpm.

### Mutant construction

Strains with *ECE1* (*ece1*Δ/Δ) gene deletion in *C*. *albicans* SC5314 were generated using the pSFS2A vector [54,55]. All of the primers used for vector construction were synthesized by MDbio Biotech Co, Inc (Taipei, Taiwan) and the sequences are available in Table 2. Briefly, the primer pairs ECE1AF/ECE1AR and ECE1BF/ECE1BR were used to respectively amplify the 5’- and 3’-flanking DNA fragments of the *ECE1* gene by polymerase chain reaction (PCR). The amplified DNA fragments were cloned into pSFS2A by restriction enzymes ApaI/XhoI and NotI/SacI to generate the pSFS-ECE1KO vector. *C*. *albicans* SC5314 was transformed with the pSFS-ECE1KO, which was linearized by ApaI/SacI, to generate heterozygous *ece1Δ*/*ECE1* strains. The *SAT1* marker was recycled under treatment with 1% (wt/vol) maltose. The heterozygous strains (*ece1Δ*/*ECE1*) were retransformed with the linearized pSFS-ECE1KO to generate the homozygous *ece1*Δ/Δ strain. The *ECE1* revertants were constructed as described below. *ECE1* endogenous promoter and open reading frame (ORF) was amplified using the primer pair ECE1FLF/ECE1FLR. The resulting products were cloned into the pSFS2A by restriction enzymes ApaI/XhoI to generate the pSFS-ECE1 plasmid. The homozygous *ece1*Δ/Δ strain was transformed with the pSFS-ECE1 plasmid, which was linearized by HpaI, to generate the *ECE1* revertant strains (SC5314 *ece1*Δ/Δ+*ECE1*). To generate candidalysin-deficient *ECE1*_Δ*184–279*_ strains, *ECE1*_Δ*184–279*_ was obtained by overlap elongation with the fragments amplified by the primer pairs ECE1FLF/ECE1D183-280OER and ECE1D280F/ECE1FLR. The DNA fragment of *ECE1*_Δ*184–279*_ was cloned into PCR-BluntII-TOPO (Thermo Fisher Scientific) for sequence confirmation, and subcloned into the pSFS2A by restriction enzymes ApaI/XhoI to generate the pSFS-ECE1_Δ*184–279*_ plasmid. The homozygous *ece1*Δ/Δ strain was transformed with the pSFS-ECE1_Δ*184–279*_ plasmid, which was linearized by HpaI, to generate the *ECE1*_Δ*184–279*_ revertant strains deficient in candidalysin peptide Ece1-III_62-93_ production (SC5314 *ece1*Δ/Δ+*ECE1*_Δ*184–279*_).

**Table 2:** Primers used in this study.

Primer	Sequence
ECE1AF	GGAGCGGGGCCCTTTCTGGAGTAATCCTATTGTTCGC
ECE1AR	GGAGCGCTCGAGTATGTAAGATTTGTGGGCGGG
ECE1BF	GGAGCGGCGGCCGCTTTGGTGTCTCTTTGCGTGTAAA
ECE1BR	GGA GCGGAGCTCCAATCTTGTCGTGCCACTGATTA
ECE1FLF	GGAGCGGGGCCCCACGCCTGTTGTTGGCAAATG
ECE1FLR	GGAGCGCTCGAGCTGCTGAGCATTTAAGCTTTTCCG
ECE1D183-280OER	CCAGCAACAACAGAATCAATATCTTCTCTTTTGGTAATAGCAGTATTGAATTCT
ECE1D280F	GAAGATATTGATTCTGTTGTTGCTGG
ECE1QF	TGCCATTTGTTGTCAGAGCTG
ECE1QR	TAGCTTGTTGAACAGTTTCCAGG
EFG1QF	ATCACAACCAGGTTCTACAACCAAT
EFG1QR	CTACTATTAGCAGCACCACCC
NRG1QF	GGTTGCACGTTGTCGAAACC
NRG1QR	TGTTGCTGCTGCTGCTTGG
YWP1QF	TGCTAGTACTGC TAACAAAGTCAC
YWP1QR	CACCATTAACACCACCAGCA
HWP1QF	GCTGGTTCAGAATCATCCA TGC
HWP1QR	AAGGTTCAGTGGCAGGAGCTG
ALS3QF	CAACTTGGGTTATTGAAACAAAAACA
ALS3QR	AGAAACAGAAACCCAAGAACAACCT
HGC1QF	GTCAGCTTCCTGCACCTCATC
HGC1QR	AAACAGCACGAGAACCAGCG
PHR2QF	ACACTGACGCTTCTGCTTTCG
PHR2QR	GCAGCTTCGTCTTCACCACA
ACT1QF	AAGAATTGATTTGGCTGGTAGAGA
ACT1QR	TGGCAGAAGATTGAGAAGAAGTTT

The sequence for overlap extension PCR is underlined.

### Histochemistry staining

The catheters were fixed in 4% paraformaldehyde, cut into 5-mm long and embedded with Leica TP1020 Automated Benchtop Tissue Processor (Leica). After embedding, the tissues were sectioned into 10-μm thick slices (RM2125 RTS, Leica), and affixed onto slides. Hematoxylin and Eosin (H&E) staining and Periodic Acid-Schiff (PAS) staining were performed with the kits procured from CIS-biotechnology.

### Fluorescence staining

The catheters were fixed in 4% paraformaldehyde, cut into 5-mm long, followed by incubation in the blocking buffer (0.4% Triton-X 100 with 1% BSA in PBS) for overnight at 4°C. For staining of fungal cells and fungal biofilms on coverslips in *in vitro* culture, the samples were fixed in 4% paraformaldehyde followed by incubation in the blocking buffer (0.4% Triton-X 100 with 1% BSA in PBS) for 1 hour at room temperature. The catheters and the cells were stained with the following antibodies and chemicals: BV421 mouse anti-human CD45 (1:100, 563879, BD Biosciences), rabbit anti-*Candida* (1:1000, ab53891, Abcam), rat anti-mouse Ly6G (1:500, 551459, BD Biosciences), hFc-mDectin-1a (1:1000, fc-mdec1a, Invivogen), Alexa Fluor 594 AffiniPure Goat Anti-Human IgG (H+L) (1:1000, Jackson ImmunoResearch), Calcofluor white (10μg/mL, BU-29067, Biotium), rhodamine-conjugated Concanavalin A (Con A) (1:500, RL-1002, Vector Laboratories), anti-rabbit antibody conjugated with Alexa Flour 594 (1:500, Invitrogen), Alexa Fluor 488 phalloidin (1:500, Invitrogen), and rabbit anti-Histone H3 (citrulline R2 + R8 + R17) (1:1000, ab5103, Abcam). The coverslips containing *C*. *albicans* biofilms were gently mounted using 10 uL of Fluoromount-G mounting medium (SouthernBiotech) before observation under a microscope.

### Gene expression analysis

Overnight culture of *C*. *albicans* yeast cells at 28°C in YPD was adjusted to the OD_600_ 0.5 and cultured in complete RPMI culture media (RPMI [Simply] with 10% FBS [Biological Industries]) or the total parenteral nutrition Oliclinomel N4-550E Emulsion for Infusion (Baxter) at 37°C with shaking at 180 rpm. Total RNA extraction of *C*. *albicans* cells was performed with the Monarch Total RNA Miniprep Kit (New England Biolabs). The concentration and purity of the extracted RNAs were determined using a Nanodrop 2000 spectrophotometer (Thermo Scientific). First-strand cDNA synthesis was conducted using the HiScript III TM First Strand cDNA Synthesis Kit (Bionovas Biotechnology), and the reaction was carried out in a MyCycler Thermal Cycler (Bio-Rad). The synthesized cDNAs were served as a template for quantitative PCR using the Luna Universal qPCR Master Mix kit (New England Biolabs) and the StepOnePlus Real-Time PCR system (Applied Biosystems by Life Technologies). The sequences of the primers for the gene expression quantification are listed in Table 2. The gene expression was quantified with the ΔCT method relative to the expression of *ACT1* in each sample.

### Mouse neutrophil isolation

Mouse neutrophils were isolated as described with modifications [56–58]. Mouse bone marrow cells were obtained from femurs and tibias of 7-week old C57BL/6 mice provided by National Laboratory Animal Center (NLAC), NARLabs, Taiwan. The procedure was approved by the Institutional Animal Care and Use Committee of National Yang Ming Chiao Tung University. The mouse bone marrow cells were resuspended in RPMI supplemented with Antibiotic-Antimycotic at 2x concentration (Simply Biologics). The cell suspension was then passed through a 40 μm mesh filter (BD Bioscienc), followed by incubation with RBC lysis buffer (Invitrogen) to lyse the red blood cells. Next, the residual living cells were suspended in 45% Percoll solution (Cytiva Percoll PLUS Centrifugation Media). Percoll gradients were prepared by layering 2 mL each of the 52 and 62% Percoll solutions on top of 3 mL of 81% Percoll solution in a conical 15-mL centrifuge tube. The bone marrow cell suspension was slowly applied to the top to the Percoll gradients and centrifuged at 1000 ×g for 30 minutes with no brake at 25°C. The cells located in the layer 3 (between 62% and 81% Percoll) within the Percoll gradient were collected and washed with HBSS (Invitrogen). Cell viability was validated by trypan blue (Invitrogen) and propidium iodide (PI) (Invitrogen) staining. Mouse Ly6G staining (rat anti-mouse Ly6G, 551459, BD Biosciences) was performed to validate the purity of the isolated neutrophils.

### Biofilm formation assay

Overnight culture of *C*. *albicans* yeast cells at 28°C in YPD was adjusted to the OD_600_ 0.5 and resuspended in complete RPMI culture media (RPMI [Simply Biologics] with 10% FBS [Biological Industries]) or the total parenteral nutrition Oliclinomel N4-550E Emulsion for Infusion (Baxter). The cells were cultured in a 24-well cell culture plate, in which each well contained a coverslip pre-coated with 1% FBS. The culture was incubated at 37°C for 24 hours without shaking to develop mature biofilms, followed by removal of unbound fungal cells by three times of PBS wash. The effect of neutrophils and candidalysin on *C*. *albicans* biofilms was performed by adding 2 x 10^5^ mouse bone marrow neutrophils and 15 μM of candidalysin peptide (Ece1-III_62-93_, SIIGGIIMGILGNIPQVIQIIMSIVKAFKGNKR, synthesized by Genomics, Taiwan, purity 95.21%) to the mature biofilms in the culture environment as indicated for another 6 hours at 37°C [20]. The coverslips were then washed with PBS followed by fixation with 4% paraformaldehyde and staining.

### Image analyses

The images of the unstained catheters, the fungal cells in different culture media, and the biofilms on the coverslips were captured using a Zeiss Axio Observer 7 fluorescence microscope. The images of H&E or PAS stained samples were captured using an Olympus BX63 microscope and processed by the Olyvia (Ver. 2.9, Olympus Life Science). The two-photon images were obtained by a Zeiss LSM 7MP multiphoton microscope and processed by ZEN (blue edition, Ver. 3.0, Zeiss). The 3D images of the catheters were reconstituted by Imaris (Bitplane). The volume of citrullinated Histone H3 in catheters was quantified by measuring the relative volume of anti-citH3^+^ signals in individual stacked images (327 μm ⊆ 327 μm ⊆ 80 μm for each stack) and analyzed with Imaris (Bitplane).

### Statistical analysis

All data analyses were performed using Prism V8 (GraphPad). Statistics was carried out using the two-tailed unpaired Student T test, or the one-way ANOVA test followed by Tukey’s multiple comparison test or Dunnett’s multiple comparison test.

## Supporting information

S1 Fig Expression of *ALS3*, *HGC1* and *PHR2* in the *C*. *albicans* catheter isolates cultured in RPMI and TPN. *C*. *albicans* yeast cells were cultured in RPMI (A) or TPN (B) at 37°C for 24 hours. Gene expression was quantified by qRT-PCR and normalized to *ACT1* expression. Data represent mean with SD from two independent experiments. Statistical analysis was performed with the one-way ANOVA, followed by Dunnett’s multiple comparison test as compared to the reference strain SC5314 (A and B) or a Student *T* test (C). *, P<0.05. **, P<0.01. NS, no significant difference.
(PDF)

S2 Fig Diverse biofilm properties of the catheter isolates and the interaction of fungal biofilms with mouse neutrophils in RPMI. *C*. *albicans* cells were cultured in RPMI for 24 hours to allow biofilm formation on a coverslip. The biofilms developed in RPMI were then incubated with mouse bone marrow-derived neutrophils (BMN) in RPMI in the absence or presence of candidalysin (CaL, 15 μM) for another 6 hours before fixation and staining. BMN NETosis was observed by Ly6G (cyan), F-actin (white) and citrullinated histone H3 staining (C-H3, red). *Candida* cells were stained by mDectin-1-Fc (green) that recognizes β-glucan on fungal cell wall. (A) Representative images are shown. (B) *Candida* biofilms were quantified as the area of *C*. *albicans* cells in each field on the coverslips. (C) The percentage of NETotic BMNs (C-H3^+^ Ly6G^+^ cells/ total Ly6G^+^ cells) in the biofilms is shown. All the images were analyzed with Imaris (Bitplane). (B-C) Results shown are mean with SD from 3 independent experiments, 2 images from each coverslip. Statistical analysis was performed with one-way ANOVA, followed by a Tukey’s multiple comparison test (B) or a Student *T* test (C). *, P<0.05. **, P<0.01. ***, P<0.001. ****, P<0.0001. NS, no significant difference. Scale bar, 50 μm.
(PDF)

S1 Data Excel spreadsheet containing, in separate sheets, the underlying numerical data for Figs 4B, 6A, 6B, 7B, 7C, 7D, 7E, 8B, 8C, 8D, 8E, S1A, S1B, S1C, S2B and S2C. (XLSX)
