# Supplementary figures and images for: Regulation of candidalysin underlies Candida albicans persistence in intravascular catheters by modulating NETosis
Source: PLoS Pathog. 2024 Jun 17;20(6):e1012319. doi: 10.1371/journal.ppat.1012319 (PMC11213320; doi:10.1371/journal.ppat.1012319)

FIG S1

A

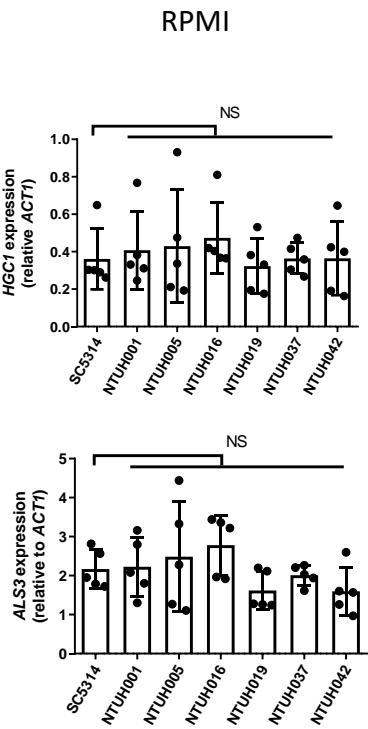

B

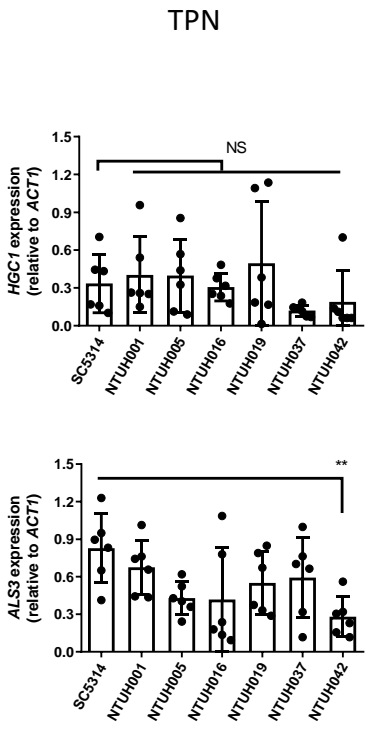

C

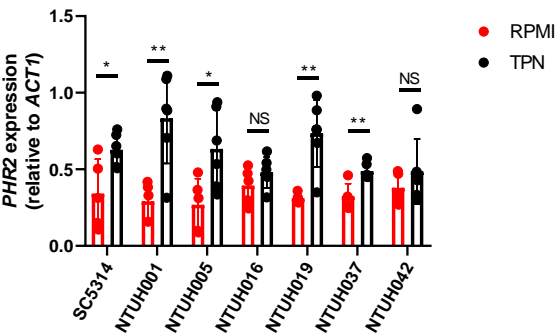

Supplement: S1 Fig — C. albicans yeast cells were cultured in RPMI (A) or TPN (B) at 37°C for 24 hours. Gene expression was quantified by qRT-PCR and normalized to ACT1 expression. Data represent mean with SD from two independent experiments. Statistical analysis was performed with the one-way ANOVA, followed by Dunnett’s multiple comparison test as compared to the reference strain SC5314 (A and B) or a Student T test (C). *, P<0.05. **, P<0.01. NS, no significant difference. (PDF) [file ppat.1012319.s001.pdf]

FIG S2  
A

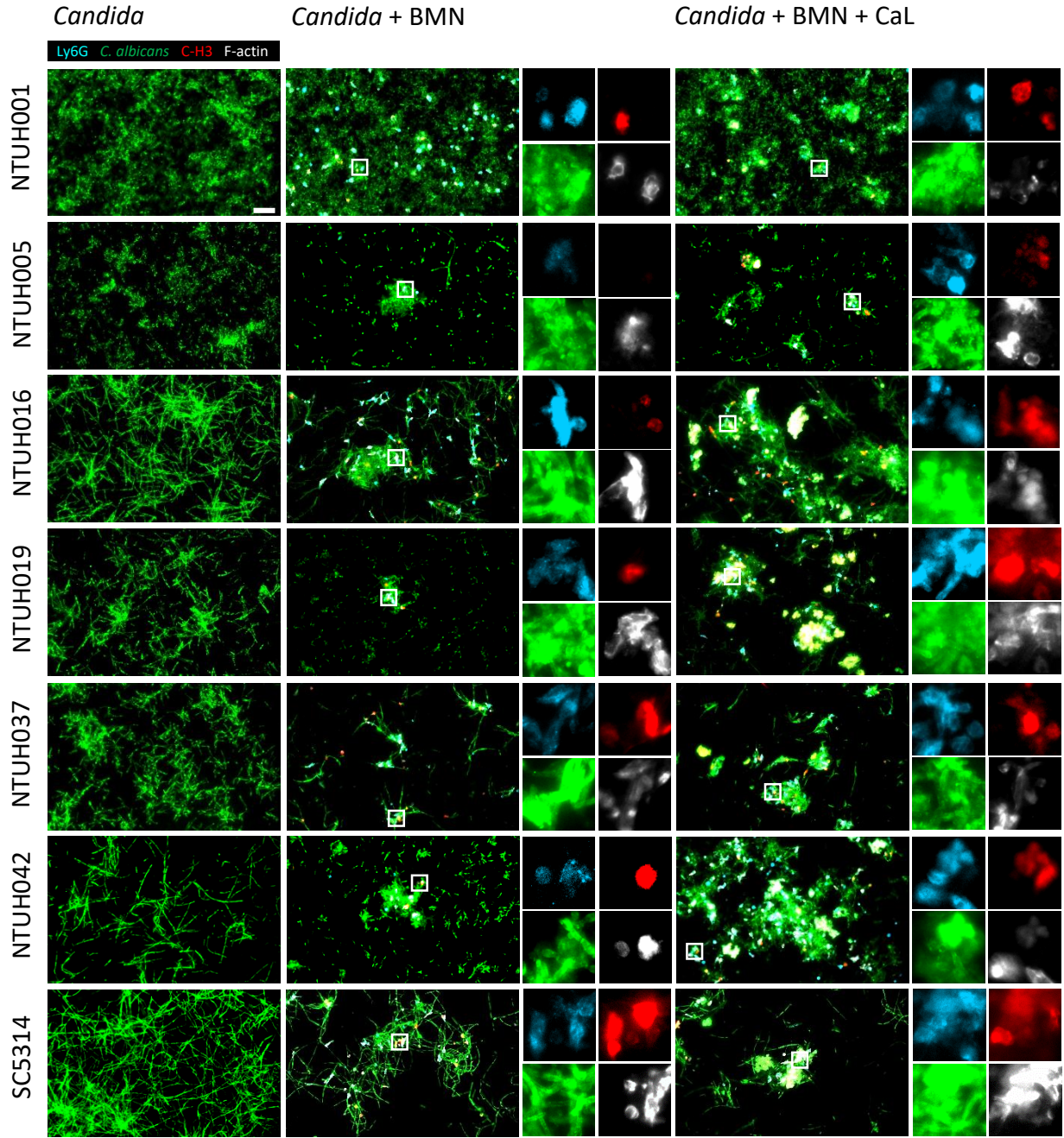

B

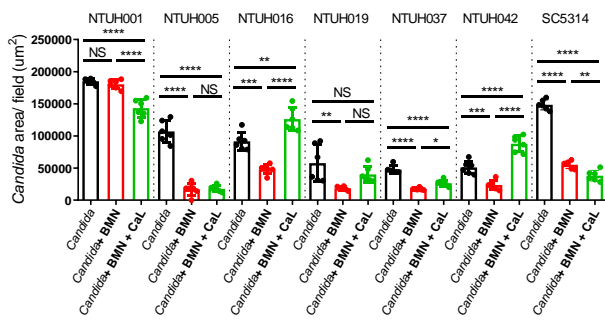

C

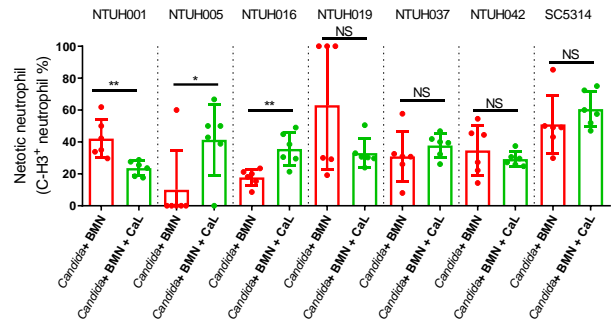

Supplement: S2 Fig — C. albicans cells were cultured in RPMI for 24 hours to allow biofilm formation on a coverslip. The biofilms developed in RPMI were then incubated with mouse bone marrow-derived neutrophils (BMN) in RPMI in the absence or presence of candidalysin (CaL, 15 μM) for another 6 hours before fixation and staining. BMN NETosis was observed by Ly6G (cyan), F-actin (white) and citrullinated histone H3 staining (C-H3, red). Candida cells were stained by mDectin-1-Fc (green) that recognizes β-glucan on fungal cell wall. (A) Representative images are shown. (B) Candida biofilms were quantified as the area of C. albicans cells in each field on the coverslips. (C) The percentage of NETotic BMNs (C-H3+ Ly6G+ cells/ total Ly6G+ cells) in the biofilms is shown. All the images were analyzed with Imaris (Bitplane). (B-C) Results shown are mean with SD from 3 independent experiments, 2 images from each coverslip. Statistical analysis was performed with one-way ANOVA, followed by a Tukey’s multiple comparison test (B) or a Student T test (C). *, P<0.05. **, P<0.01. ***, P<0.001. ****, P<0.0001. NS, no significant difference. Scale bar, 50 μm. (PDF) [file ppat.1012319.s002.pdf]
